# Effects of *acuC* on the growth development and spinosad biosynthesis of *Saccharopolyspora spinosa*

**DOI:** 10.1186/s12934-021-01630-2

**Published:** 2021-07-22

**Authors:** Zhudong Liu, Jie Xiao, Jianli Tang, Yang Liu, Ling Shuai, Li Cao, Ziyuan Xia, Xuezhi Ding, Jie Rang, Liqiu Xia

**Affiliations:** State Key Laboratory of Development Biology of Freshwater Fish, Hunan Provincial Key Laboratory for Microbial Molecular Biology, College of Life Science, Hunan Normal University, Lushan Road 36, Changsha, 410081 China

**Keywords:** *Saccharopolyspora spinosa*, Acetoin utilization protein, Spinosyns, Acetylation/deacetylation posttranslational modification

## Abstract

**Background:**

Acetoin utilization protein (acuC) is a type I histone deacetylase which is highly conserved in bacteria. The *acuC* gene is related to the acetylation/deacetylation posttranslational modification (PTM) system in *S. spinosa.* Spinosyns, the secondary metabolites produced by *Saccharopolyspora spinosa*, are the active ingredients in a family of insect control agents. However, the specific functions and influences of acuC protein in *S. spinosa* are yet to be characterized.

**Results:**

The knockout strain and overexpression strain were constructed separately with the shuttle vector pOJ260. The production of spinosyns A and D from *S. spinosa-acuC* were 105.02 mg/L and 20.63 mg/L, which were 1.82-fold and 1.63-fold higher than those of the wild-type strain (57.76 mg/L and 12.64 mg/L), respectively. The production of spinosyns A and D from *S. spinosa-*Δ*acuC* were 32.78 mg/L and 10.89 mg/L, respectively. The qRT-PCR results of three selected genes (*bldD*, *ssgA* and *whiA*) confirmed that the overexpression of *acuC* affected the capacities of mycelial differentiation and sporulation. Comparative proteomics analysis was performed on these strains to investigate the underlying mechanism leading to the enhancement of spinosad yield.

**Conclusions:**

This study first systematically analysed the effects of overexpression *acuC* on the growth of *S. spinosa* and the production of spinosad. The results identify the differentially expressed proteins and provide evidences to understand the acetylation metabolic mechanisms which can lead to the increase of secondary metabolites.

**Supplementary Information:**

The online version contains supplementary material available at 10.1186/s12934-021-01630-2.

## Background

*Saccharopolyspora spinosa* is a gram-positive bacterium that is non-acid-resistant and aerobic [1]. Spinosyns are synthesized by fermentation of *S. spinosa* under aerobic conditions [2], and they are novel macrolide compounds with high insecticidal activity and can be widely used in crop pests such as *Coleoptera*, *Hymenoptera,* and *Diptera* [3]. It is a promising biopesticide which has strong insecticidal activity and broad pesticidal spectrum. The most active and abundant spinosyns from *S. spinosa* fermentation media are spinosyns A and D. The mixture of spinosyn A (85% of spinosad) and spinosyn D (15% of spinosad) is called spinosad, which has no negative effects and low toxicity on nontarget insects and mammals compared with traditional chemical pesticides [4–6]. The 74-kb spinosyn biosynthetic gene cluster contains 23 open reading frames (ORF) including five genes encoding type I polyketide synthase (PKS) (*spnA*, *B*, *C*, D, *E*); four genes involved in the intramolecular C–C bond formation (*spnF*, *J*, *L*, *M*); four genes responsible for rhamnose attachment and methylation (*spnG*, *I*, *K*, *H*); six genes involved in forosamine biosynthesis (*spnP*, *O*, *N*, *Q*, *R*, *S*); four genes involved in spinosyn branched chain biosynthesis and glycosylation modification (*gtt*, *gdh*, *epi* and *kre*). *S. spinosa* CCTCC M206084 isolated by our lab in southern China has the ability to produce spinosad. However, the yield of spinosad is too low to meet the needs of industrial production. The fermentation period is relatively long and restricts the production of spinosad [7]. The activity of Acs is modulated by acetylation/deacetylation system, which is critical to the synthesis of the acetyl-AMP intermediate from acetate and ATP. The overexpression of *acuC* affects the gene expressions related with the mycelial differentiation and sporulation in *S. spinosa* as well as the production of spinosad.

The acetylation levels of proteins are controlled by an acetylase and a deacetylase. The acetylase catalyzes the acetyl group transferred from acetyl-CoA to the substrate protein during translation [8]. However, the deacetylase has the opposite function, that removes the acetyl group from acetylated protein. Two types of deacetylases are involved in this process. Zinc-dependent (mainly histone deacetylases) and NAD-dependent deacetylases play a notable role in the metabolic process.

In 1996, the first histone deacetylase was found [9, 10]. Histone acetylation/deacetylation occurs in the genetic material of natural organisms and serves as an important biological information regulator extensively. Histone deacetylases exist widely in plants, animals, microorganisms, archaebacteria and eubacteria [11]. Currently, HDACs are classified into two families: the NAD-dependent sirtuin family and the traditional HDAC family [12].

The acetoine utilization protein (*acuC*) is a class I deacetylase which only exists in bacteria. The acetylation of acetyl-CoA synthetase of *S. coelicolor* is regulated in vivo, but the acetyl transferase that is responsible for its acetylation has not been identified [13, 14]. This protein is highly conserved in *Streptomycetes*, sharing 75–91% amino acid sequence identities, especially for the DNA binding domain. In *Bacillus subtilis*, the regulation of deacetylases is more complex. There are two main types of deacetylases: the deacetylase SrtN of the NAD-dependent Sirt family and the deacetylase *acuC* of the NAD-independent family [15]. *acuA* is a homologous gene of acyltransferase identified in *B. subtilis* [16, 17]. According to the comparison of amino acid sequence of Translated BLAST in NCBI website, we found that the amino acid sequence of acuC protein in *S. spinosa* had 79.38% similarity to that of *B. subtilis*. The query cover of these two sequences was 89%.

To investigate the effects of *acuC* on the mycelial differentiation and spinosad biosynthesis, the fragment of *acuC* was cloned from the genome of *S. spinosa* by PCR amplification, and the *acuC* gene was simultaneously placed under the strong promoter *P*_*ermE*_ [18]_._ Then, the knockout vector pOJ260-Δ*acuC* and the overexpression vector pOJ260-P_*ermE*_-*acuC* were constructed by digestion and ligation. The knockout vector pOJ260-Δ*acuC* was introduced into *S. spinosa* genome by conjugative transfer to block the expression of the *acuC* gene. The overexpression vector pOJ260-P_*ermE*_-*acuC* was introduced into the *S. spinosa* genome by conjugative transfer to overexpress *acuC* with the same method [19].

In this study, homologous recombination technology was used to place the integrative vector regulated under a strong promoter. The overexpression of *acuC* influenced the spinosad biosynthesis and mycelial differentiation. To gain insight into the molecular mechanism underlying this phenomenon, we compared the differential proteins, the insecticidal activity and the glucose consumption in the wild-type and mutant strains. The results showed that the overexpression of *acuC* led to great changes in gene expression, thereby affected the mycelial differentiation and sporulation. The spinosad biosynthesis, phenotypes and insecticidal activities of mutant strains were also influenced by the overexpression of *acuC*.

## Results

### Effects of knockout and overexpression of *acuC* on morphology and sporulation

**Fig. 1:**
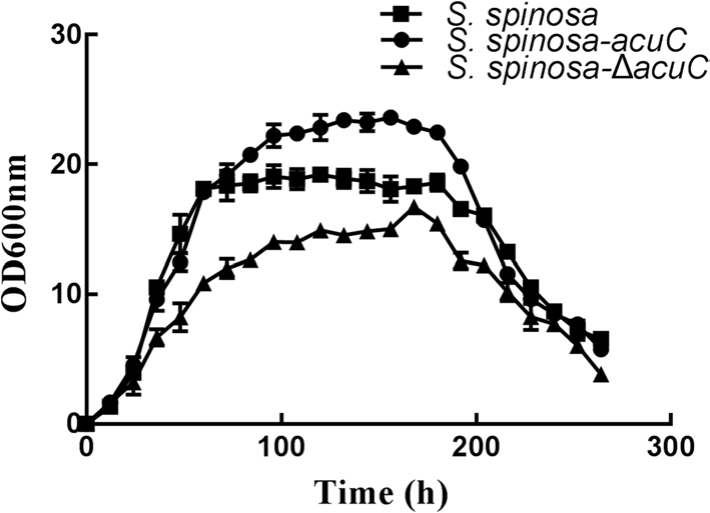
Growth curve of *S. spinosa, S. spinosa*-Δ*acuC* and *S. spinosa*-*acuC*

The growth characteristics of the wild-type and mutant strains were measured in fermentation medium (Fig. 1). The results showed that the logarithmic growth phases of *S. spinosa* and *S. spinosa-acuC* were basically synchronous, and the wild-type and *S. spinosa-acuC* strains entered the stationary growth phase after 60 h. However, the biomass of *S. spinosa-acuC* at the stationary growth phase was higher than that of the wild-type strain. The *S. spinosa-*Δ*acuC* strain entered the stationary growth phase at 84 h and entered the death phase after 200 h. The biomass of the *S. spinosa-*Δ*acuC* strain was significantly lower than that of the wild-type strain.

**Fig. 2:**
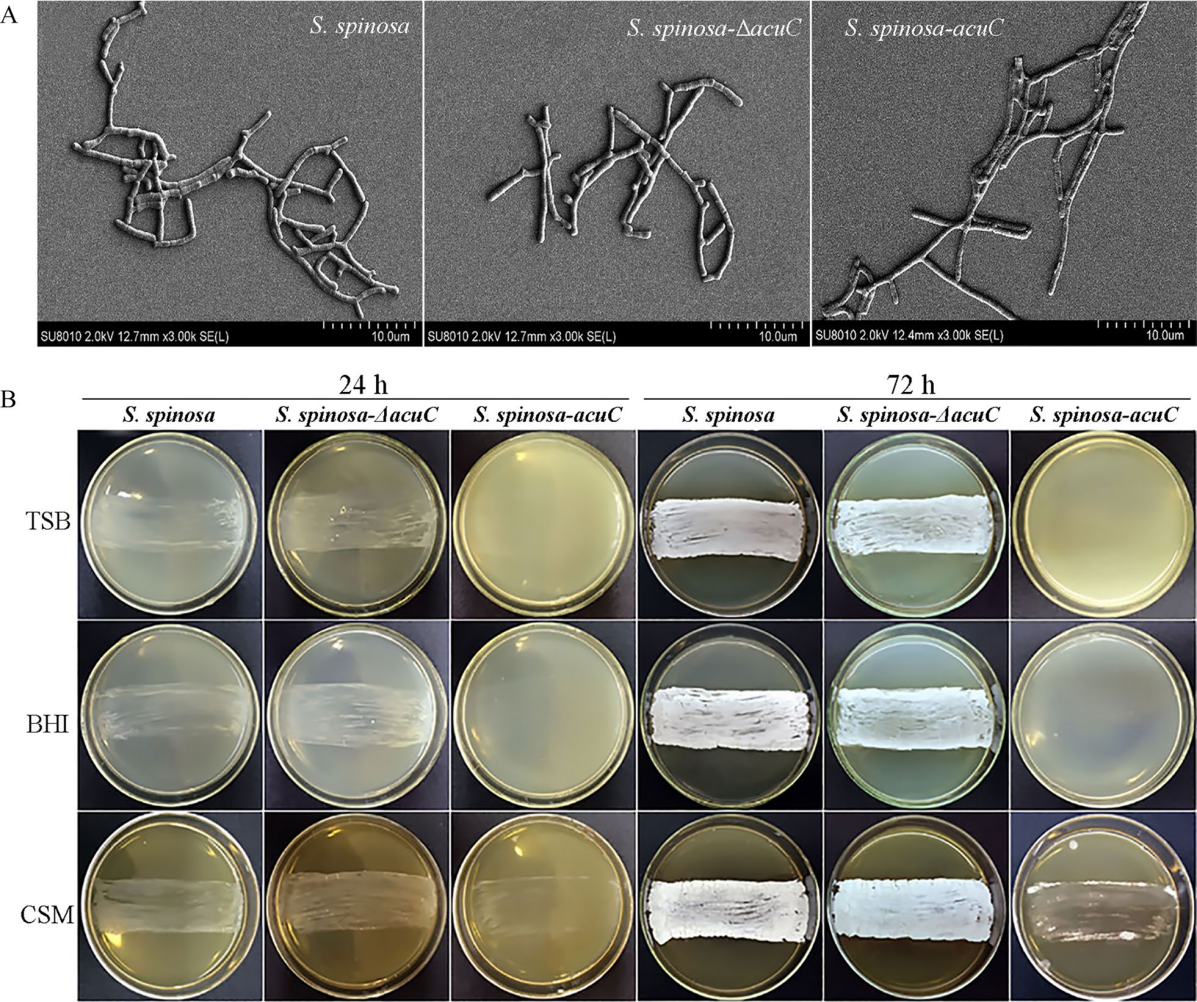
Effects of *acuC* on the morphology and sporulation of *S. spinosa, S. spinosa-*Δ*acuC* and *S. spinosa-acuC.* **A** Scanning electron morphological observation of wild-type and mutant strains after 48 h; **B** The morphological comparison of wild-type and mutant strains on different solid media. The sporulation capacities of *S. spinosa-*Δ*acuC* and *S. spinosa* in three culture media were obviously stronger than that of *S. spinosa-acuC*. The *S. spinosa-acuC* produced only a few spores on CSM medium and almost no spores were produced on TSB and BHI medium

These strains were cultured in CSM medium and collected at 48 h. After the samples were dehydrated, the morphology of mycelium and development of the wild-type and mutant strains were observed by scanning electron microscopy (SEM). The results showed that the mycelium of *S. spinosa-*Δ*acu*C was slender and more shrunken than that of the wild-type strain and the hyphal length of *S. spinosa-*Δ*acu*C was longer (Fig. 2A). However, the mycelia of *S. spinosa-acu*C were plumper and more fragmented than those of the wild-type strain.

**Fig. 3:**
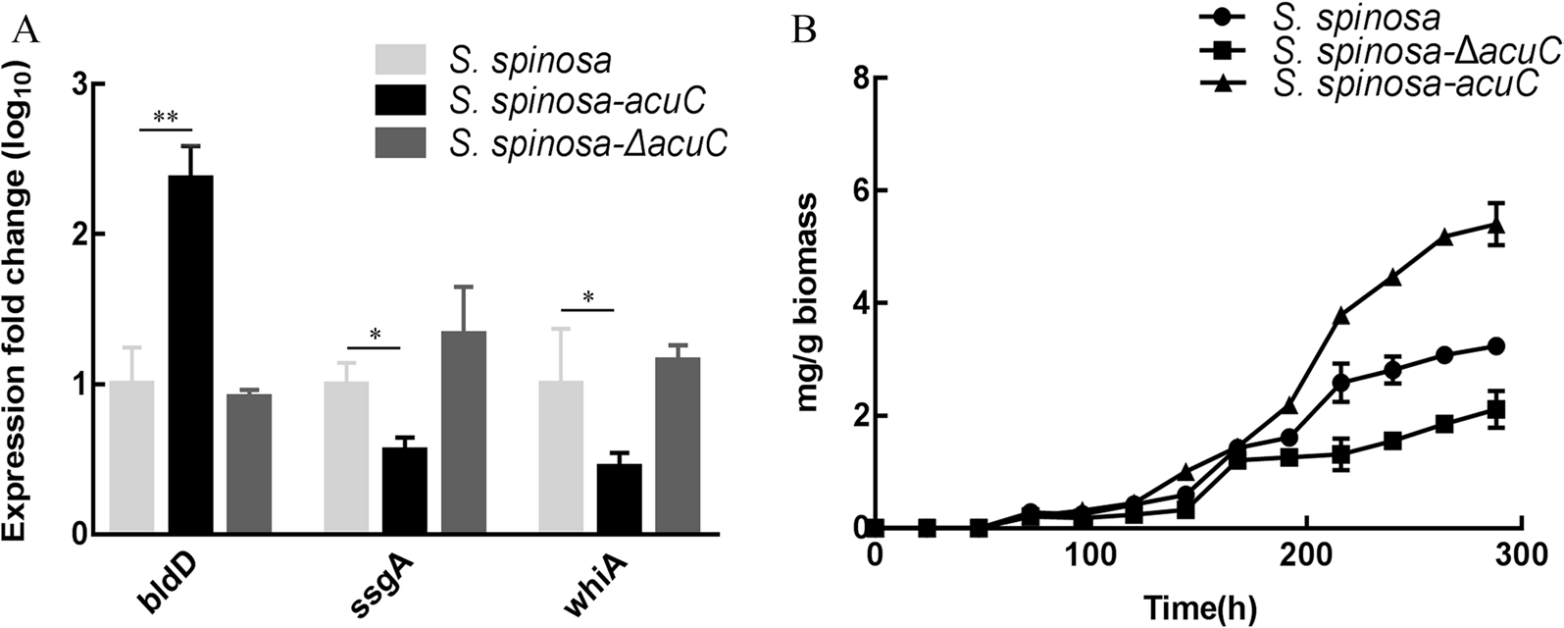
Expression levels of *ssgA*, *whiA* and *bldD* and spinosad production related to biomass in *S. spinosa, S. spinosa-*Δ*acuC* and *S. spinosa-acuC.* **A** The mRNA samples were isolated from the wild-type and mutant strains on 72 h, the expression of *bldD* exhibited a significant up-regulated in *S. spinosa-acuC*, whereas *whiA* and *ssgA* was down-regulated. The 16S rRNA gene was used as an internal control to quantify the relative expression of the target genes. Gene expression differences are shown by the bar height. Error bars indicated the standard errors of results from n = 3 replicates. * and ** indicated *P* < *0.05* and *P* < *0.01*, respectively. **B** The process development formation rate of spinosad related to biomass in *S. spinosa, S. spinosa-*Δ*acuC* and *S. spinosa-acuC*

The sporulation in different solid culture media between the wild-type and mutant strains were observed on BHI, TSB and CSM solid media (Fig. 2B). The growth and sporulation of *S. spinosa* on these three different solid media are different due to their different components. The sporulation capacity of *S. spinosa-*Δ*acuC* and *S. spinosa* were obviously stronger than that of *S. spinosa-acuC* on these three solid media. The spores of *S. spinosa-acuC* were produced only a bit on the CSM medium and were hardly produced on TSB and BHI media. This phenomenon lasted the whole incubation period. The expression level of *whiA* and *ssgA*, the sporulation related genes in *S. spinosa-acuC* was downregulated, but the expression of *bldD,* a mycelial differentiation related gene was upregulated in *S. spinosa-acuC* (Fig. 3A). The results demonstrated that *acuC* affected the expression of *whiA*, *bldD* and *ssgA* and also explained the low sporulation of *S. spinosa-acuC.*

During the fermentation of streptomycetes, controlling the differentiation of mycelial to maintain the proper length and branching to avoid clumping are the key factors in increasing yield of secondary metabolites. The growth mode of *S. spinosa* is filamentous growth. Spores are dormant bodies produced by spore-producing microorganisms when the external environment lacks nutrients. In Fig. 2B, we found that the spores of *acuC* overexpression strain were significantly different from those of wild-type and knockout strain on three different media. Therefore, the overexpression of *acuC* affected the sporulation to some extent and further affected the biosynthesis of spinosad.

### Effects of *acuC* knockout and overexpression on spinosad biosynthesis

**Fig. 4:**
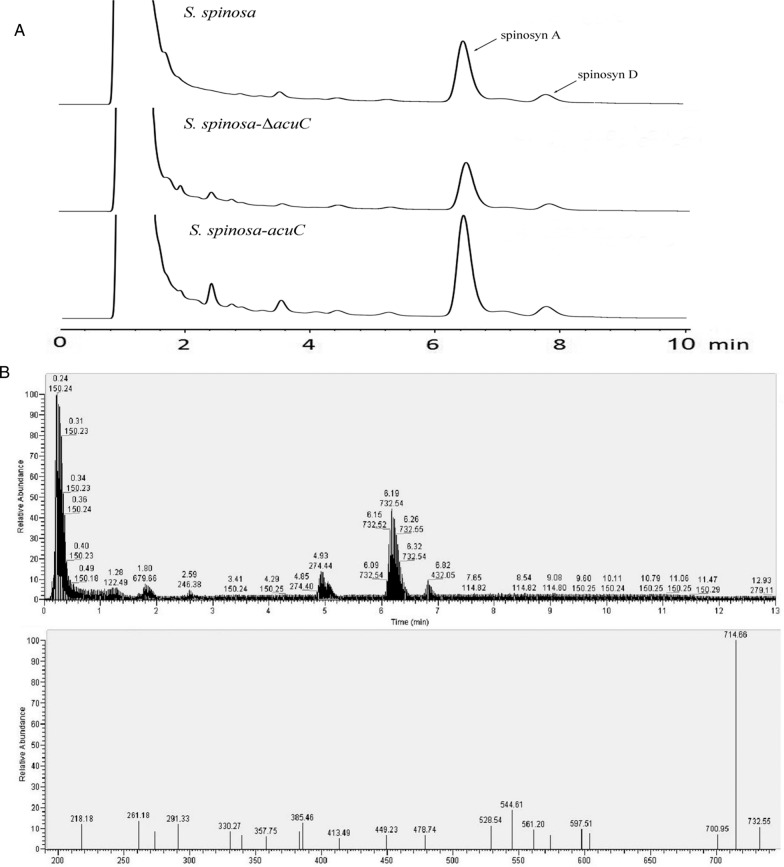
The yield of spinosad between the wild-type and mutant strains. **A** The HPLC analysis of fermentation yield products of the wild-type and mutant strains; **B** The mass spectrometry identification of the target peak

By analysing the consumption of glucose content in the fermentation medium during the growth process, we found that the overexpression of *acuC* accelerated the consumption of glucose (Additional file 1: Figure S6). Compared with the wild-type (0.083 g/L/h) and knockout strain (0.076 g/L/h), the glucose consumption rate of *acuC* overexpression strain (0.093 g/L/h) was improved. The spinosyns standard sample was tested with different concentration gradients, and the standard curve of spinosyn concentration (mg/L) with the peak areas (mAU*s) was obtained: y = 0.2504x + 3.4532, R^2^ = 0.9991. Spinosyns A and D were extracted and measured from the fermentation broths of the wild-type and mutant strains by HPLC (Fig. 4A). The production of spinosyns A and D from *S. spinosa-acuC* were 105.02 mg/L and 20.63 mg/L, which was 1.82-fold and 1.63-fold higher than those of the wild-type strain (57.76 mg/L and 12.64 mg/L), respectively. In addition, *S. spinosa-*Δ*acuC* produced only a small amount of spinosad. The production of spinosyns A and D from *S. spinosa-*Δ*acuC* were 32.78 mg/L and 10.89 mg/L, respectively. We measured the production of spinosad and the biomass of wild-type and mutant strains for 288 h to observe the process development formation rate of spinosad related to biomass (Fig. 3B). The spinosad production rate of *S. spinosa*-acuC strain (0.0188 mg/g/h) was faster than that of the wild-type strain (0.0112 mg/g/h) and the knockout strain (0.0073 mg/g/h). According to the objective comparison of spinosad production rate and the glucose consumption rate, the overexpression of *acu*C has a certain effect on the production of spinosad. The target peaks of fermentation products were collected for mass spectrometry identification. The MS results showed the [M + H]^+^ ions at *m/z* = 732.5 (Fig. 4B). This observation verified that the substance was the main component of spinosad [6].

### Biological insecticidal activity of the wild-type and mutant strains

The insecticidal activities of the wild-type and mutant strains were assayed by comparing the half lethal time (LT_50_) values. The half lethal time (LT_50_) of *S. spinosa-acuC* was 0.435 d less than that of *S. spinosa* (Table 1). On the first day, the survival number of *H. armigera* was basically the same between *S. spinosa* and *S. spinosa-acuC*, and the survival rates were similar. From the next day, the *S. spinosa-acuC* fermentation exhibited stronger toxicity to *H. armigera* than the wild-type strain fermentation. The survival rate of *S. spinosa-*Δ*acuC* was higher than that of wild-type and *S. spinosa*-*acuC* strains, suggesting that the insecticidal activity of *S. spinosa-*Δ*acuC* on *H. armigera* was weaker than that of the wild-type strain (Additional file 1: Figure S3). These results showed that the insecticidal effect of *S. spinosa-acuC* was significantly higher than that of the wild-type strain and the knockout strain had the worst insecticidal effect.

**Table 1:** Biological insecticidal activity of the wild-type and mutant strains

Strains	Relative coefficient (R^2^)	LT_50_(d)	95% Confidence interval
*S. spinosa*	0.9911	4.093	3.729–4.527
*S. spinosa*-Δ*acuC*	0.9812	4.534	4.153–5.087
*S. spinosa*-*acuC*	0.9877	3.658	3.333–4.036

### Significantly differential proteins influenced by the overexpression of *acuC*

**Fig. 5.**
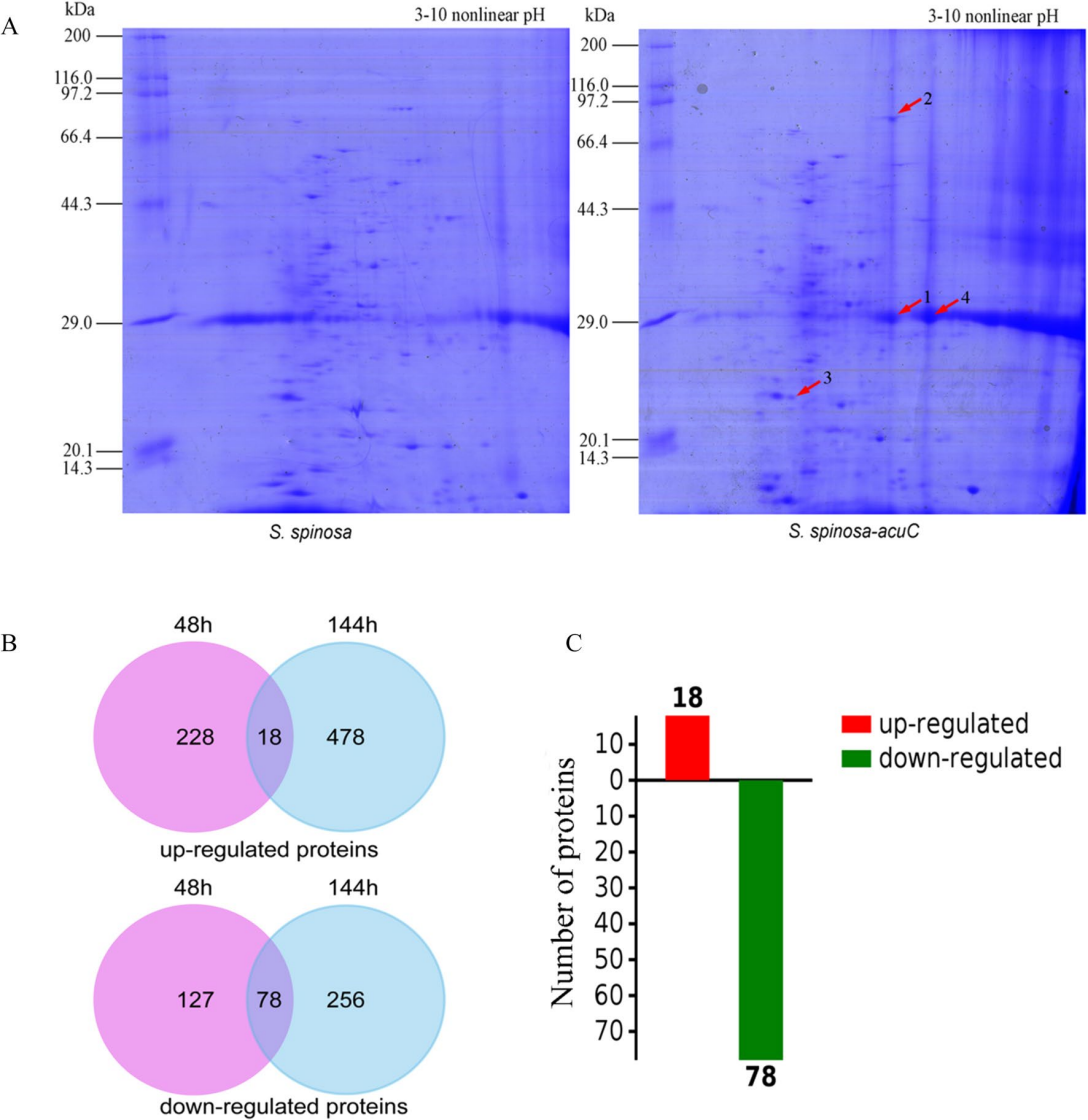
2D-gel electrophoresis and semi-quantitative proteomics analysis of the wild-type and mutant strains. **A** 2D-gel electrophoresis analysis of differential proteins between *S. spinosa* and *S. spinosa*-*acuC*; **B** Numbers of the differential proteins between *S. spinosa* and *S. spinosa-acuC* in 48 h and 144 h; **C** Up-regulated and down-regulated proteins between *S. spinosa* and *S. spinosa-acuC*

The whole-cell proteins of the wild-type and two mutant strains were analysed by SDS-PAGE at 48 h. The results showed that large numbers of differential proteins were found in the lane of the over-expression strain (Additional file 1: Figure S7). In order to further separate and identify these differential proteins, we performed 2D-gel electrophoresis analysis between the wild-type strain and *S. spinosa*-*acuC*. After the first-dimension isoelectric focusing, the samples from the equilibrated IPG strips were transferred to the second-dimension (Fig. 5A). To obtain optimal separation and identification, the differential proteins selected by 2D-gel electrophoresis were measured by 1D-LC–MS/MS. The identified proteins were categorized by UniProt (www.uniprot.org) and KEGG (www.kegg.jp). We analysed the metabolic processes involved in these identified proteins (Table 2). Four differential proteins, *infB, paaZ, serA* and *acuA* were found, which were associated with the hydrolysis of GTP, synthesis of L-serine, the oxidoreductase activity and post translational modification of acetylase/deacetylase.

**Table 2:** The differential proteins identified by 2D-gel electrophoresis analysis

Band number	Uniprot number	Protein description	Gene	Theoretical MW (KDa)	Possible function
1	A0A2N3Y983	Tanslation initiation factor IF-2	*infB*	28.69	Hydrolysis and promotes its binding to the 30S ribosomal subunits. Also involved in the hydrolysis of GTP during the formation of the 70S ribosomal complex
2	A0A2A3Z889	bifunctional aldehyde dehydrogenase/enoyl-CoA hydratase	*paaZ*	72.0	The oxidoreductase activity, acting on the aldehyde or oxo group of donors, NAD or NADP as acceptor
3	A0A2N3Y9R3	D-3-phosphoglycerate dehydrogenase	*serA*	23.34	This protein is involved in step 1 of the subpathway that synthesizes L-serine from 3-phospho-D glycerate
4	A0A0F6JEE7	acetoin utilization protein acetyltransferase AcuA	*acuA*	30.32	This protein is involved to be an acetyltransferase of the class I histone deacetylases (HDACs)

Then, semiquantitative proteomics samples were prepared and analysed from the wild-type and overexpression strains. In Fig. 5B, the light purple circle represents the fold change value in the overexpression strain compared with the wild-type strain at 48 h (228 upregulated proteins and 127 downregulated proteins), and the light blue circle represents the fold change value of the overexpression strain compared with the wild-type strain at 144 h (478 upregulated proteins and 356 downregulated proteins). The intersection of the two sets of data represents the common differential proteins (18 upregulated and 78 downregulated) of the wild-type and overexpression strains. Thresholds of 1.5- fold and 0.75- fold were used to indicate significantly upregulated and downregulated protein expression respectively. Moreover, 18 upregulated proteins and 78 downregulated proteins were discovered (Fig. 5C).

### GO enrichment and KEGG pathway analysis affected by the overexpression of *acuC*

**Fig. 6:**
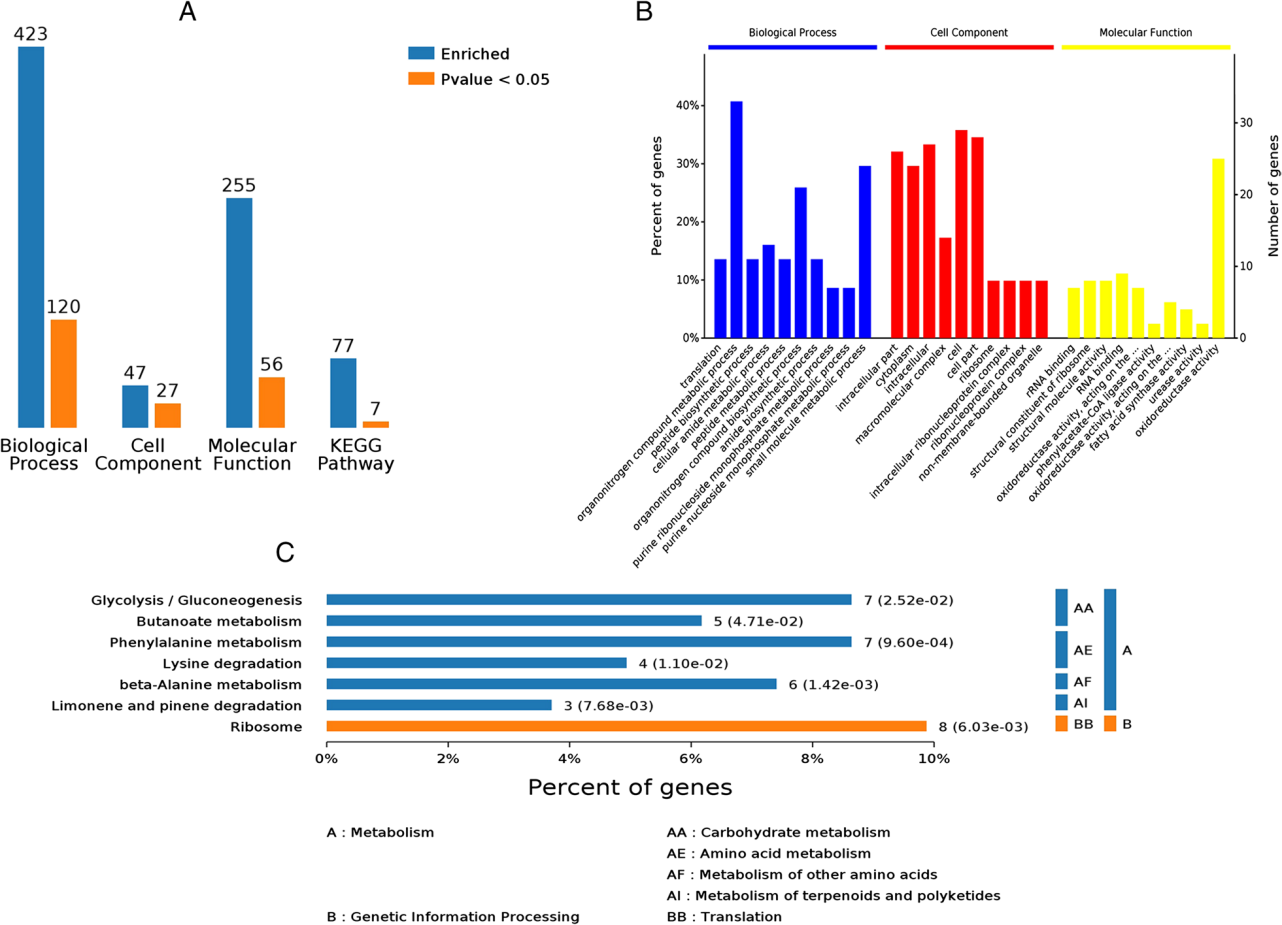
GO enrichment and KEGG pathway analysis of difference proteins. **A **GO enrichment number analysis of proteins; **B** The percent statistics of GO enrichment analysis; **C** Classes of enriched KEGG Pathway. The GO enrichment analysis and KEGG pathway analysis revealed that significant difference proteins mainly involved in the glycolysis/gluconeogenic, phenylalanine and β-Alanine metabolic pathways

The significantly differential proteins were subjected to GO enrichment analysis of the functional and metabolic pathways. First, in the classification of biological pathways, 423 pathways were detected in GO enrichment assays, and 120 biological pathways (*P* < *0.05*) were detected with significant differences (Fig. 6A); these genes were mostly involved in post translational modification, the regulation of organic nitrogen compound metabolism and small molecule metabolism. Second, in the classification of cell component, 47 pathways were detected in GO enrichment assays, and 27 pathways (*P* < *0.05*) were detected with significant differences; these were mostly involved in the transformation of intracellular components, the cytoplasm and cellular components. Finally, in the category of molecular function, 255 pathways were detected in GO enrichment assays, and 56 pathways (*P* < *0.05*) were detected with significant differences; these were mostly involved in molecular changes related to the main redox enzyme activities. The overexpression of *acuC* caused obvious changes in biological processes and intracellular molecular function (Fig. 6B). The GO enrichment and KEGG pathway analysis of different genes revealed that differential proteins were mainly involved in the glycolysis/gluconeogenic, phenylalanine and β-alanine metabolic pathways (Fig. 6C). The functions of the significantly upregulated genes are shown in Table 3, and these genes were involved in energy metabolism, amino acid metabolism and the secondary metabolite biosynthesis. The overexpression of *acuC* affects the post transcriptional modification system and the biosynthesis of secondary metabolites.

**Table 3:** The types of differential genes related to spinosad biosynthesis

ID	Gene	Protein name	Fold change	Identity
497996893	*efp*	Elongation factor P	1.53	88.83
498001352	*nusG*	Transcription termination/antitermination protein	1.87	85.71
498001860	*purH*	Bifunctional purine biosynthesis protein	1.56	89.06
498381195	*pepB*	Group B oligopeptidase	1.61	83.47
497997129	*iolG3*	Inositol 2-dehydrogenase 3	1.65	86.6
498381383	*fabH*	3-oxoacyl-[acyl-carrier-protein] synthase	1.94	83.89
498378866	*fabG1*	3-oxoacyl-[acyl-carrier-protein] reductase	1.95	88.13
497991096	*atpD*	ATP synthase subunit beta	1.98	96.7
497998623	*fba*	Fructose-bisphosphate aldolase	2.03	91.28
498381238	*nadE*	NH(3)-dependent NAD(+) synthetase	3.03	81.48
498381374	*ahpE*	Alkyl hydroperoxide reductase E	0.31	91.45
497992400	*ftsZ*	Cell division protein	0.38	62.5
497994003	*clpC*	Negative regulator of genetic competence	0.73	91.45

## Discussion

This study used knockout and overexpression methods to explore the function of *acuC* in *S. spinosa* for the first time [18], and protoplast transformation method was used to integrate recombinant plasmids into the genome of *S. spinosa*. To a certain extent, the method overcomes the complicated restriction modification system in actinomycetes. The structure and biosynthesis of spinosyn analogues have been clearly described [20], and studies on knocking out the key genes and the improvement of heterologous expression have been studied [21]. Chaoyi Song et al. constructed a spinosyn artificial gene cluster that contains 7 operons with constitutive promoters and compared with the original gene cluster. The mutant gene cluster caused a 328-fold increase of spinosyn production in *Streptomyces albus* J1074 [22]; Jie Rang et al. deleted a hybrid NRPS-T1PKS biosynthetic gene cluster via Latour gene knockout system in *Saccharopolyspora pogona*. The deletion of this gene cluster enhanced the butenyl-spinosyn biosynthesis and growth development [23]. On the basis of our research, AcuC deacetylase maintained the balance between free CoA and acetyl-CoA during acetoin catabolism in *S. spinosa*. These precursors could flow steadily into the biosynthesis of secondary metabolites.

The acetylation of histone proteins is common in the growth and development of organisms. This phenomenon plays an important role in the regulation of gene expression and posttranslational modification of proteins. The *acuC* is part of the acuABC operon and related to the acetyl-CoA synthetase. We searched the bacteria contained acuC protein sequence in UniProt. Then these amino acid sequences were compared and analysed by MEGA 5.0 software. The same color of the amino acid residues in sequences indicated that the amino acid residues from the N-terminus of the proteins were conserved (Additional file 1: Figure S8). Therefore, we analysed the functional impact of *acuC* in *S. spinosa*. By comparing the content of acetyl-CoA between the wild-type and overexpression strains in 48 h, 96 h and 192 h (Additional file 1: Figure S4), we found that the overexpression of *acuC* had an important regulatory effect on the accumulation and consumption of secondary metabolite precursors. These results indirectly regulate the growth cycle of bacteria, metabolic processes and the biosynthesis of secondary metabolites in *S. spinosa*.

The expression of *acuC* affects the acetylation/deacetylation system, which changes the contents of the spinosyn biosynthesis precursors. The detection of spinosad production by HPLC revealed that the yields of spinosyns A and D from *S. spinosa-acuC* were 1.82-fold and 1.63-fold higher than those of the wild-type strain [24], whereas *S. spinosa-*Δ*acuC* produced less spinosad than the wild-type strain. The analysis of differential proteins between the wild-type and *S. spinosa*-*acuC* found 18 significantly upregulated proteins by semiquantitative proteomics analysis. The functions of these upregulated proteins were analysed by UniProt and KEGG. The nadE is an NH_3_-dependent NAD^+^ synthetase which has the highest fold change. The high expression of this protein affects the enzyme activity in the secondary metabolite biosynthesis and energy metabolism. Two upregulated proteins were identified in amino acid metabolism. purH is a bifunctional purine biosynthetic protein involved in purine metabolism, and pepB is an oligopeptidase which degrades peptides. Three upregulated proteins associated with energy metabolism were found: fabH, a 3-oxoacyl-acyl-carrier-protein synthase involved in the synthesis and metabolism of fatty acids and catalyzed the condensation reaction of fatty acid synthesis by adding two carbonyl acyl acceptors of malonyl-ACP; fabG, a 3-oxoacyl-acyl-carrier-protein reductase, involved in the biosynthesis of fatty acids; atpD, participated in the oxidative phosphorylation, was accompanied by the production of ATP from ADP in the presence of a proton gradient on the membrane (Table 3).

In this study, the mycelia of *S. spinosa-acu*C were plumper and more fragmented than those of the wild-type strain. The gene expression level of mycelial differentiation related gene *bldD* was significantly increased in the overexpression strain. The expression of *whiA* is related to the synthesis of the white pigment characteristic of mature *S. venezuelae* spores [25]. The *ssgA* was confirmed to be involved in the regulation of the white phenotype [26]. The effects of *bldD* on mycelial differentiation, cell growth and the secondary metabolites production have been reported in previous studies [27, 28].

In addition, according to the semiquantitative proteomics analysis, 78 significantly downregulated proteins were found in *S. spinosa-acuC*. Three proteins were involved in the regulation of growth and development. The *ahpE* gene encodes alkyl hydroperoxide reductase E, which acts as an antioxidative stress regulator in cell protection by detoxifying peroxisomes, but the expression of this gene is significantly decreased in *S. spinosa-acuC*. The *ftsZ* gene encodes an essential cell division protein which forms a contractile ring structure (Z ring) at future cell division sites. The regulation of ring assembly controls the timing and location of cell division. The ftsZ ring recruits other cell division proteins in the septum to produce a new cell wall between the dividing cells, which regulates cell growth and death. The *clpC* gene encodes the ATP-dependent Clp protease ATP-binding subunit, which is a necessary regulator for the growth of cells and organisms at high temperature. The results of the semiquantitative proteomics proved that the overexpression of *acuC* reduced the sporulation ability on solid medium.

**Fig. 7:**
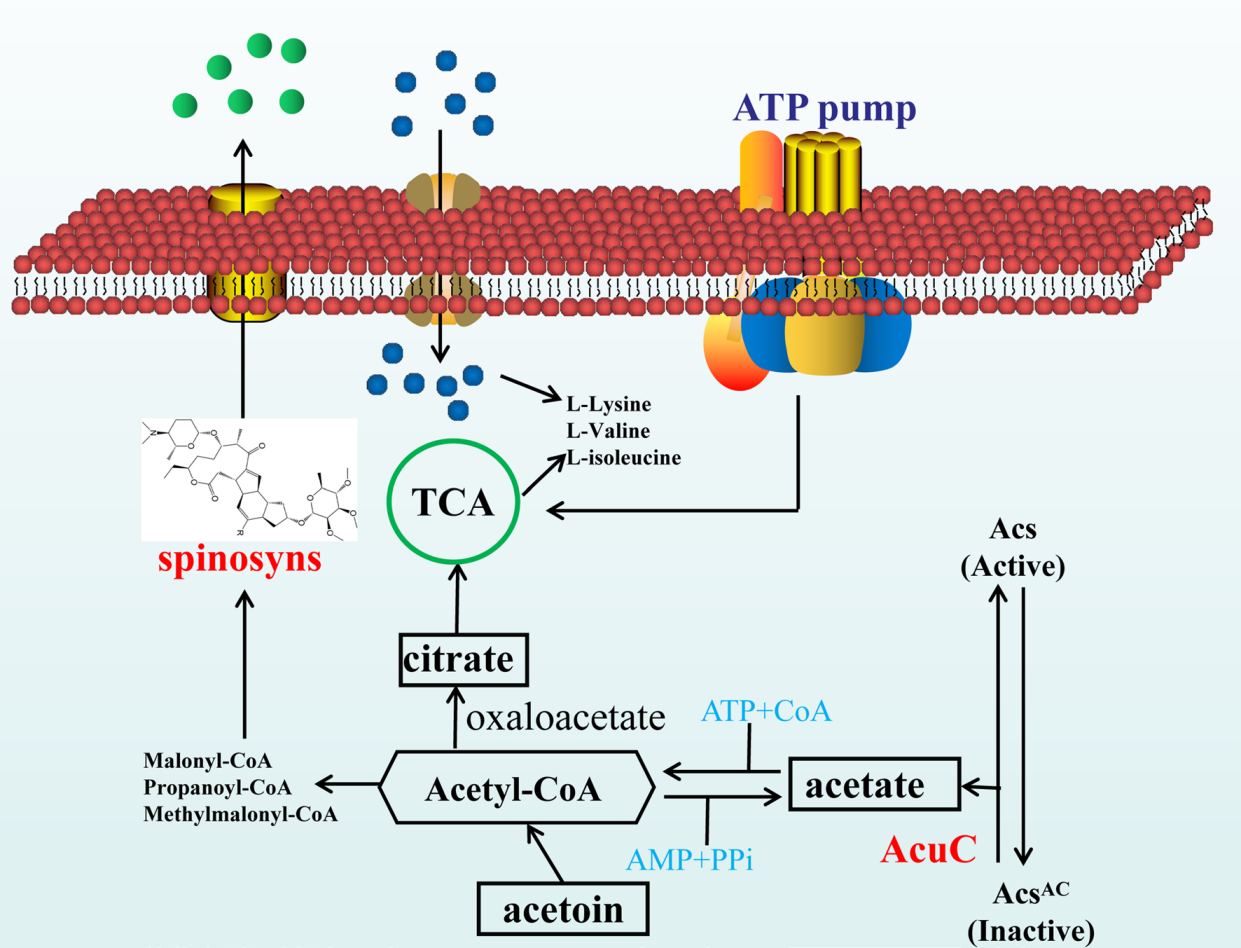
Regulatory network schematic diagram of AcuC enzyme on acetoin catabolism. The overexpression of *acuC* activates the acetylated Acs protein, promotes the production of acetate and the catalysis from acetate to acetyl-CoA, and provides numbers of precursors for spinosad biosynthesis

Then, we analysed the role of *acuC* in acetoin catabolism pathway. The measurement of acetyl-CoA contents showed that the content of acetyl-CoA in the overexpression strain increased significantly in 96 h (Additional file 1: Figure S4). The overexpression of *acuC* caused the activation of acetylated Acs protein and promoted the production of acetate, which further affected the biosynthesis of acetyl-CoA and provided precursors for spinosad biosynthesis (Fig. 7). According to the results of acetate contents remaining in bacteria (Additional file 1: Figure S5), the accumulation rate of acetate content in *acuC* overexpression strain (0.095 mM/L/h) was higher than that in wild-type strain (0.077 mM/L/h) during the logarithmic phase. However, during the stationary phase, the accumulation rate of acetate in the overexpression strain (0.040 mM/L/h) began to slow down, comparing with the wild-type strain (0.107 mM/L/h). The overexpression of *acuC* promoted the catalysis from acetate to acetyl-CoA during the stationary phase and provided precursors for spinosad biosynthesis.

Our study enhanced the current understanding of the metabolic and developmental functions of *acuC* via knockout and overexpression. The overexpression of *acuC* promoted the mycelial differentiation and the catalysis from acetate to acetyl-CoA in the stationary phase, which led to an increase of spinosad production.

## Conclusions

The function experiments of knocking out and overexpression of *acuC* showed that this gene significantly affected the mycelial differentiation, sporulation and morphology in *S. spinosa*. After overexpression of *acuC* in *S. spinosa,* the production of spinosyns A and D in *S. spinosa-acuC* were 105.02 mg/L and 20.63 mg/L, which were 1.82-fold and 1.63-fold higher than the wild-type strain (57.76 mg/L and 12.64 mg/L), respectively. The insecticidal activity of *S. spinosa-acuC* was significantly improved. The function of *acuC* was identified as a key role in the acetoin catabolism pathway based on the proteomics analysis and the measurement of precursors between the wild-type and mutant strains. The strategy based on the proteomics analysis provides evidences to understand the acetylation metabolic mechanisms and improve the production of secondary metabolites in streptomycetes.

## Methods

### Strains, plasmids and growth conditions

The strains and plasmids used in this study are listed in Additional file 1: Table S1, The primers are listed in Additional file 1: Table S2. The spores of *S. spinosa* were cultivated in CSM activation media (10 g/L glucose; 45 g/L trypticase soy broth; 9 g/L yeast extract; 2.2 g/L MgSO_4_^.^7H_2_O) in a 300 mL flask, with a starting volume of 50 mL at 30 °C with 300 rpm. After cultivation of the strains for 48 h, 3 mL bacteria solution add to 30 mL fementation media (1.0 g/L KNO_3_; 0.01 g/L FeSO_4_; 0.4 g/L K_2_HPO_4_^.^3H_2_O; 0.4 g/L MgSO_4_^.^7H_2_O; 20 g/L glucose; 4 g/L yeast extract; 4 g/L tryptone; pH = 7.2) and incubated at 30 °C with 300 rpm. The culture condition of the *S. spinosa-*Δ*acuC* and *S. spinosa-acuC* is the same as the wild-type strain with antibiotic in medium (apramycin, 50 mg/L). The conjugative transfer and protoplast transformation between *E. coli* and *S. spinosa* by using R6 medium (200 g/L sucrose; 10 g/L Dextrin; 26 g/L BHI; 1 g/L Casamino acid; 0.1 g/L K_2_SO_4_; 0.05 g/L FeSO_4_^.^7H_2_O; 0.05 g/L MgSO_4_^.^7H_2_O; 0.001 g/L MnCl_2_^.^4H_2_O; 0.001 g/L ZnSO_4_^.^7H_2_O; 0.01 mol MOPS; 0.048 mol CaCl_2_; 0.065 mol L-glutamic acid; 2% agar powder). Then incubated at 30 ℃ for 5–7 days. The yield of spinosad was detected in the initial fermentation medium by HPLC (60 g/L Glucose; 5 g/L Bean cake powder; 7 g/L corn syrup; 22.5 g/L cottonseed meal; 10 g/L soluble starch; 2 g/L yeast powder; 20 mL butyl oleate; 5 g/L CaCO_3_; 2.5 mL trace elements; pH = 7.2–7.4), and incubated at 30 °C with 300 rpm, 10–12 d. The spore morphology of *S. spinosa* was observed in BHI (38 g/L BHI, 2% agar powder), CSM and TSB (tryptic soy broth medium) and incubated at 30 °C for 6 days. All strains of *E. coli* were grown in lysogeny broth medium at 37 °C supplemented with antibiotic as required (apramycin, 50 mg/L).

### Construction and verification of the mutant strains

To produce the knockout vector pOJ260-Δ*acuC*, we amplified the *acuC* sequence from the genome of *S. spinosa* CCTCC M206084 with the primer pair *acuC*-F and *acuC*-R (Sangon, Shanghai, China). The fragment was digested with *Hin*dIII and *Eco*RΙ and ligated into the corresponding restriction sites of pOJ260. Recombinant vector was constructed by T4 ligase-linking the fragment and pOJ260 after the *Hin*dIII- and *Eco*RΙ- enzyme digestion (Additional file 1: Figure S1). To confirm the successful knockout of the *acuC* fragment, we amplified *Apr* and *acuC* fragments in the mutant strains genomes with the primer pairs *Apr*-F/*Apr*-R and *acuC*-F/*acuC*-R (Additional file 1: Figure S1).

The *P*_*ermE*_ gene was amplified by the primer pair *perm*-F/*perm*-R from pOJ260-*cm-P*_*ermE*_ and the *acuC* gene was amplified by the primer pair *acuC*-F/*acuC*-R from the genome of *S. spinosa.* The fragments were fused by overlap extension PCR by primer pair *perm*-F/*acuC*-R. Recombinant vector pOJ260-*P*_*ermE*_-*acuC* was constructed by T4 ligase-linking the fused fragment and pOJ260 after the *Hin*dIII- and *Eco*RV-enzyme digestion (Additional file 1: Figure S2). To confirm the pOJ260-*P*_*ermE*_-*acuC* was successfully inserted into the chromosome, we amplified the fragment in the mutant strain genome with the primer pair *P*_*ermE*_-F and *acuC*-R. The wild-type genomic and pOJ260-*P*_*ermE*_-*acuC* were used as the positive and negative controls, respectively (Additional file 1: Figure S2).

### Cultivation profile analysis and measurement of glucose in wild-type and mutant strains

To monitor the growth profiles of wild-type and mutant strains, cells were collected randomly for the growth curve measurement. The cell concentration during fermentation was measured by absorbance at 600 nm (OD_600_). The glucose contents in the supernatants were measured by 3,5-nitrosalic acid (DNS) reagent. The absorbance of different samples were measured at 540 nm by spectrophotometer. After 48 h of cultivation in CSM medium, 40 μL of wild-type and mutant strains were streaked on BHI, CSM and TSB solid media respectively and incubated at 30 °C for 5 days. The main components of Trypticase Soy Broth (TSB) are tryptone, soy protease digestion, NaCl, KH_2_PO_4_ and glucose. The main components of Brain Heart Infusion (BHI) are beef brain dip powder, beef heart dip powder, tryptone, casein tryptone, NaCl, glucose and Na_2_HPO_4_. The main components of CSM are glucose, yeast extracts and MgSO_4_·7H_2_O. The cell morphology was observed by Hitachi SU8010 cold field scanning electron microscopy [29]. The wild-type and mutant strains were cultivated for 48 h in CSM activation media. Then these strains were transferred to 50 mL synthetic fermentation media and cultivated to 288 h. 500 uL fermentation supernatants of the wild-type and mutant strains were extracted with the equal volume of ethyl acetate for 1 h. The supernatant was evaporated and added 50 μL methanol, centrifuged at 10,000 rpm for 5 min. The ethyl acetate layer was identified by HPLC (Agilent 1290, wavelength: 250 nm, C18 column: AQ12S05-1546, YMC, Japan) [30]. Each sample was loaded onto a C18 column and eluted with the elution buffer at 1.0 mL/min. The elution buffer A: 10% (v/v) acetonitrile; The elution buffer B: 90% (v/v) acetonitrile [31].

### Insecticidal activity, the measurement of acetyl-coenzyme A (Ac-CoA) and acetate content in the wild-type and mutant strains

Fermentation supernatants of the wild-type and mutant strains (1 mL) were added into 20 mL of feed (per liter: 40 g of yeast extract, 70 g of bean flour, 5 g of vitamin C, 15 g of agar, 36% acetic acid, and 10 g of penicillin; whisked until smooth and creamy). The biological activity of the cell culture liquid was measured in 24-well culture plates with one *H. armigera* worm per well; these were cultured at 30 °C with three repeats in parallel, and the mortality rate was determined every 24 h. The acetyl-CoA content was determined in total fractions using the Acetyl-Coenzyme A Assay Kit (MAK039-1KT, Sigma-Aldrich) according to the manufacturer’s instructions. The sample was diluted with a reaction mix. And fluorescence was measured using a plate reader and the following settings: λex = 535 nm and λem = 587 nm. An acetyl-CoA standard curve was made in the range of 10–1000 pmol, and the correlation coefficient was 0.990. The concentrations of the standard solutions used to generate the standard curve were 25 pmol/L, 50 pmol/L, 100 pmol/L, 200 pmol/L, and 400 pmol/L. Acetate content was quantified using the Acetate Colorimetric Assay Kit (BioVision, Milpitas, CA, USA) according to the manufacturer’s instructions. The supernatant was filtered through a 0.45 μm filter membrane after centrifugation at 14,000 rpm for 5 min and then stored at − 20 °C. All samples were analysed by 96-well fat bottom plates (Costar, Corning, NY, USA) following the kit protocol with the absorption maximum at 450 nm in Molecular Devices SpectraMax M5 Microplate Reader (Molecular Devices, Shanghai, China).

### Protein extraction, Two-dimensional electrophoresis and SDS-PAGE analysis

Cells were harvested (9000 rpm, 10 min, 4 °C) at 48 h from the wild-type and mutant strains washed four times with PBS (10 mM, pH = 7.8, precooled at 4 °C). The cell pellet was then resuspended in 200 μL of lysozyme (100 mg/mL), 600 μL of lysis buffer was added to each tube, and ultrasonic fragmentation (JY92 ultrasonic cell grinder, Ningbo new Chi biotechnology company) was used to extract the whole cell protein. Lysis buffer for protein extraction contained 8 M urea, 2 M thiourea, 4% (w/v) CHAPS, 75 mM NaCl, 50 mM Tris–HCl (pH = 8.0), 2 mM phenylmethylsulfonyl fluoride and 4 μL of a protease inhibitor cocktail powder (P8465, Sigma, St. Louis, MO, USA). After quantitative analysis of the protein by Bradford assay, the samples were checked by SDS-PAGE before proceeding with LC–MS/MS analysis. For SDS-PAGE analysis, proteins from cell lysates (20 μg of total protein of each phase) were separated from 4 to 12% NuPAGE gels (Invitrogen, Carlsbad, CA, USA), stained with Coomassie brilliant blue (CBB) for 3 h and destained overnight [32].

Two-dimensional electrophoresis culture sample was decanted and 1 mL of lysis buffer (9 mol/L UREA, 4% CHAPS, 1% IPG buffer, 1% DTT) was added. The sample was disrupted by ultrasonication for 5 min and centrifuged (12,000 *g* for 20 min at 4 °C) to remove the precipitate [33]. Next, 1 mL of pre-cooled acetoine was added and the sample was kept at –20 °C overnight. The supernatant was removed after centrifugation (10,000 g for 30 min at 4 °C). The precipitate was dried and 500 mL of protein hydration solution was added. The extracted protein was quantified and used for two-dimensional electrophoresis. Briefly, 800 μg of the protein sample was removed, dry strips (Bio-Rad, United States) were prepared (pH 3–10 IPG), and run for the first-dimension isoelectric focusing. The equilibrated strips were placed in the gel slab for the second-dimensional sodium dodecyl sulfate–polyacrylamide gel electrophoresis. After electrophoresis, the gel was stained with Coomassie blue. Following decolorization, the gel was scanned by Microtek Bio-5000 protein scanner (Microtek, China).

### LC–MS/MS analysis

Tryptic peptides were desalted and concentrated on an Oasis HLB sample cartridge column (Waters Corporation, MA, USA). The purified peptides were dried at 37 °C by vacuum freeze drying (Thermo Fisher, San Jose, CA, USA). The LC–MS/MS analysis was performed on LTQ-XL mass spectrometer (Thermo Fisher, San Jose, CA, USA) coupled with a homemade nano-ionization source, two Finnigan quaternary pumps (a sample pump and an MS pump; LC Packings, San Jose, CA) and an autosampler (LC Packings, San Jose, CA) equipped with a two-position, ten-port valve with a 25-μL sample loop. For proteomics analysis, 20 μL of the redissolved digested peptides (50-μg samples) was first separated by an SCX column (BioBasic SCX, Thermo Fisher, San Jose, CA, USA) with ten concentration steps of ammonium chloride (0, 25, 50, 100, 150, 200, 250, 300, 500 and 1000 mM NH4Cl dissolved in a buffer containing 4% acetonitrile and 0.1% formic acid) and then further separated by an RP column (BioBasic C18, Thermo Fisher, San Jose, CA, USA) as described previously. The elution buffer A was 0.1% formic acid in double-distilled water, and the elution buffer B was 0.1% formic acid in acetonitrile. For the MS detection, the mobile phase was 98% buffer A and 2% buffer B for the first 5 min, a gradient of 45 min in 40% buffer B, a gradient of 30 min in 90% B and 20 min in 98% buffer A to re-equilibrate the columns at a constant flow rate of 150 μL/min. The LTQ-XL mass spectrometer was operated using the “instrument method” of the Xcaliber software (Thermo Finnigan, San Jose, CA, USA). The temperature of the heated capillary was 180 ℃. The full MS scan ranged from 80 m/z to 2000 m/z, followed by MS/MS analysis in the positive mode and intervening MS/MS scans on the ten most intense ions. Parent ions were selected by dynamic exclusion with a repeat count of two, a repeat duration of 30 s and an exclusive duration of 90 s.

The protein database was downloaded from the National Center of Biotechnology Information (NCBI, http://www.ncbi.nlm.nih.gov/) and contained 6690 entries of *S. spinosa*. Database searches depended on the SEQUEST search engine (Proteome Discoverer 1.3 software package, Thermo Fisher, San Jose, CA, USA).

### Proteomics semiquantification and bioinformatics analysis

Three biological replicates were identified by LC-MS [34]. PAI is defined as the number of detected peptides divided by the number of detectable peptides per protein [35]. PAI was converted to emPAI [36]. We performed Venn intersection analysis on the data of the wild-type and overexpression strains, filtered the data and chose the research direction on the basis of the intersections. Venn analysis was performed on the genes differentially expressed between wild-type and overexpression strains according to the up- and down- regulation in differential data. For the detected peptides, SEQUEST counted only the number of unique peptide ions of a protein. Detectable peptides were calculated by an online tool, IPEP (http://ipep.moffitt.org/), subjected to trypsin digestion and detected by electrospray ionization using an ion trap model with two missing cleavages [37]. Biological variability was tested by parallel analysis of three independent biological replicates. Proteins were classified into different functional categories according to the Kyoto Encyclopedia of Genes and Genomes (KEGG) pathway (http://www.genome.jp/kegg), the OmicsBean database (https://www.omicsbean.cn) and the UniProt database (https://www.uniprot.org/). All metabolic pathways of *S. spinosa* were drawn with the help of KEGG pathway maps.

### The iTRAQ (isobaric tags for relative and absolute quantitation) protein analysis

The iTRAQ reagent labeled protein samples were trypsinization and lyophilization. The isobaric tagging iTRAQ reagent was added into the protein digest (75% isopropanol) and incubated at room temperature for 2 h. 100 μL water was added into the reaction. The labeled peptide pools were then mixed together and lyophilized. The samples were dissolved in 100 μL of mobile phase A (10 mM ammonium formate, 5% acetonitrile in water, pH = 10.0), and the peptide fraction was separated by HPLC (Agilent 1100) with the following conditions: column (ZORBAX Extended-C18), detection wavelength: 215 nm, flow rate: 0.3 mL/min. Separation gradient was a linear gradient from 5 to 38% of Buffer B (10 mM ammonium formate, 90% aqueous acetonitrile, pH = 10.0) for 80 min. The eluate solution was collected every minute in the gradient and dried for LC/MS analysis. The SCIEX's ProteinPilot software (v 5.0) was used for database search and protein identification and relative quantitative analysis. The false-positive rate FDR (False Discovery Rate) was set to 1%. After de-redundancy, trypsin, depth analysis mode (Thorough), mass spectrometry mass error was 20 ppm and mass spectrometry mass error was 0.1 Da.

### RNA extraction and qRT-PCR analysis

Total RNA of *S. spinosa* CCTCC M206084, *S. spinosa*-Δ*acuC* and *S. spinosa*-*acuC* were separately isolated from broth cultured in TSB medium on the forth day using TRIzol Reagent (Invitrogen). RNA concentration and purification were determined by measuring the ratio of OD260nm to OD280nm. DNase treatment and cDNA synthesis were performed by PrimeScriptTM RT Reagent Kit with gDNA Eraser (Takara) according to the manufacturer’s instructions. The transcriptional levels of related genes were assayed on 7500 Real-Time PCR system instruments (Applied Biosystems, USA). The qRT-PCR amplification was performed by using SYBR® Permix Ex TagTM GC (Takara). Primer pairs used in qRT-PCR were developed with Primer Premier 5.0 and listed in Additional file 1: Table S2. PCR was performed at the following conditions: 3 min at 50 °C and 15 min at 95 °C, followed by 35 cycles of 20 s at 95 °C and 1.5 min at 60 °C. Melting curves were performed from 60 to 95 °C to validate the specificity of PCRs. The 16S rRNA gene was used as an internal control to quantify the relative expression of the target genes.

### Statistical analysis

The results were statistically analysed by SPSS 18.0 and *p* < *0.05* indicated that there was a significant difference between each samples.

## Supplementary Information

**Additional file 1: Table S1.** Strains and plasmids used in this study. **Table S2.** Nucleotide sequences of primers. **Figure S1.** Construction and verification of *S. spinosa*-Δ*acuC.* **Figure S2.** Construction and verification of *S. spinosa*-*acuC.* **Figure S3.** The insecticidal activity of the wild-type and mutant strains on *H. armigera.* **Figure S4.** The content of Acetyl-CoA between the wild-type and overexpression strains in 48 h, 96 h and 192 h. **Figure S5.** The acetate content remained in bacteria between the wild-type and overexpression strains during fermentation. **Figure S6.** The consumption curve of glucose content in the fermentation medium during the growth process. **Figure S7.** The SDS-PAGE analysis of whole-cell proteins of the wild-type and mutant strains at 48 h. **Figure S8.** Comparison of acuC protein sequences among different bacteria.

## Data Availability

Not applicable.

## Abbreviations

2D-LC–MS/MS: Two-dimensional liquid chromatography-tandem mass spectrometry
HPLC: High performance liquid chromatography
iTRAQ: Isobaric tags for relative and absolute quantitation
Acs: Acetyl-CoA synthetase
HDAC: Histone deacetylase acetylation/deacetylation
