# Supplementary material for: Effects of acuC on the growth development and spinosad biosynthesis of Saccharopolyspora spinosa
Source: Microb Cell Fact. 2021 Jul 22;20:141. doi: 10.1186/s12934-021-01630-2 (PMC8296664; doi:10.1186/s12934-021-01630-2)
Supplement: Supplementary file 1 — Additional file 1: Table S1. Strains and plasmids used in this study. Table S2. Nucleotide sequences of primers. Figure S1. Construction and verification of S. spinosa-ΔacuC. Figure S2. Construction and verification of S. spinosa-acuC. Figure S3. The insecticidal activity of the wild-type and mutant strains on H. armigera. Figure S4. The content of Acetyl-CoA between the wild-type and overexpression strains in 48 h, 96 h and 192 h. Figure S5. The acetate content remained in bacteria between the wild-type and overexpression strains during fermentation. Figure S6. The consumption curve of glucose content in the fermentation medium during the growth process. Figure S7. The SDS-PAGE analysis of whole-cell proteins of the wild-type and mutant strains at 48 h. Figure S8. Comparison of acuC protein sequences among different bacteria. [file 12934_2021_1630_MOESM1_ESM.docx]

**Additional file 1**

# Effects of *acuC* on the growth development and spinosad biosynthesis of *Saccharopolyspora spinosa*

Zhudong Liu^#^, Jie Xiao^#^, Jianli Tang, Yang Liu, Ling Shuai, Li Cao, Ziyuan Xia, Xuezhi Ding, Jie Rang and Liqiu Xia^*^

State Key Laboratory of Development Biology of Freshwater Fish, Hunan Provincial Key Laboratory for Microbial Molecular Biology, College of Life Science, Hunan Normal University, Changsha, China.

***Correspondence:** Liqiu Xia, Hunan Provincial Key Laboratory for Microbial Molecular Biology, State Key Laboratory of Development Biology of Freshwater Fish, College of Life Science, Hunan Normal University, Lushan Road 36, Changsha 410081, China. Tel/ Fax: +86 073188872298.

E-mail address: [xialq@hunnu.edu.cn](mailto:dingxuezhi@hunnu.edu.cn)

**Table S1 Strains and plasmids used in this study**

|  | Relative description | | Sources |
| --- | --- | --- | --- |
| Strains |  |  | |
| *E.coli* Top10 | Host for general cloning | Lab store | |
| *E.coli* S17-1 | Donor strains for conjugation | Lab store | |
| *S. spinosa* | The producer strains of *spinosad* | Lab store | |
| *S.spinosa-△acuC*  *S.spinosa-acuC* | *S. spinosa* harboring pOJ260-*acuC*  *S. spinosa* harboring pOJ260-*P_ermE_-acuC* | This work  This work | |
| Plasmids |  |  | |
| pOJ260 | *E.coli* cloning vector, containing pUC18 replicon, oriT, Apra^R^ | Lab store | |
| pOJ260-cm-*P_ermE_* | Containing *P_ermE_* sequence | Lab store | |
| pOJ260-*acuC*  pOJ260-P_ermE_ -*acuC* | *acuC* inserted into pOJ260 by *Eco*R Ⅰand *Hin*d Ⅲ  *P_ermE_*-*acuC* inserted into pOJ260 by *Eco*RⅤ and *Hin* d Ⅲ | This work  This work | |

**Table S2 Nucleotide sequences of primers**

| Primer | sequence(5′→ 3′) |
| --- | --- |
| acuC-F | GGG*AAGCTT*GGGTCGATCTCGTCCTCCC (*Hin*dⅢ) |
| acuC-R  acuC-A  acuC-B | CCG*GAATTC*GTTCGTAGATGTCCGGTTCTG (*Eco*R Ι)  CCATGCAACGGAAACACC  GAGTCCGTGCTCGGCTAC |
| P_ermE_-F | CG*AAGCT*TCTGGACTTCTAGAGCTAGCC (*Hin*dⅢ) |
| P_ermE_-R | GCATGCCGGTCGACTCTA |
| P_ermE_-*acuC*-F | CGGTTGGTAGGATCCTCTAGAGTCGACCGGCATGCCTTCCAGGTTGTCGATGACC |
| P_ermE_-*acuC*-R  Apr-F  Apr-R | AGT*GATATC*CTGTACGAGTGCGTGAAGGA (*Eco*RⅤ)  GCTCATCGGTCAGCTTCTCAAC  CTTCGCATCCCGCCTCTG |
| *whiA-*F | CCGACGGGCTGAGGTTTC |
| *whiA-*R | GTGCCCGAACAGCTCGTG |
| *ssgA*-F | CGAGGGCGACGTGACGAT |
| *ssgA*-R | AGGTTCTCGTTGCCAGGCAC |
| *bldD-*F | TCGTCGGGTCCTATGAGCG |
| *bldD-*R | TCACAACTTTGGTGGCAGGC |

Note: Restriction enzyme sites were italic, overlaping sequences were underlined.


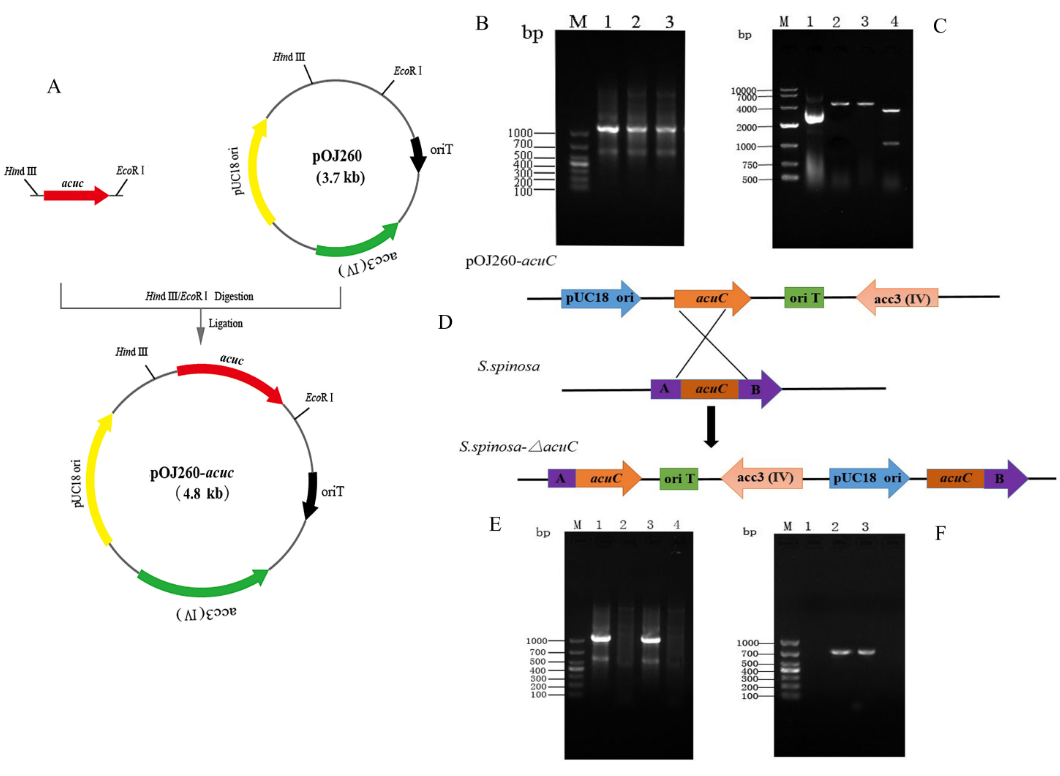


**Figure S1. Construction and verification of *S. spinosa*-Δ*acuC***

A.Construction of the gene knockout vector pOJ260-**Δ***acuC*; B. 1.1 kb *acuC* Fragment amplification by using the *S. spinosa* genome as the template and the *acuC*-F and *acuC*-R as the primer pair; C. The restriction enzyme analysis and PCR amplification of pOJ260*-*Δ*acuC;* D. The schematic diagram of *acuC* gene knockout; E. PCR validation of 1.1 kb *acuC* fragment with the primer pair *acuC*-A and *acuC*-B; F. PCR validation of Apra resistance gene around 720 bp with the primer pair Apr*-*F and Apr*-*R.


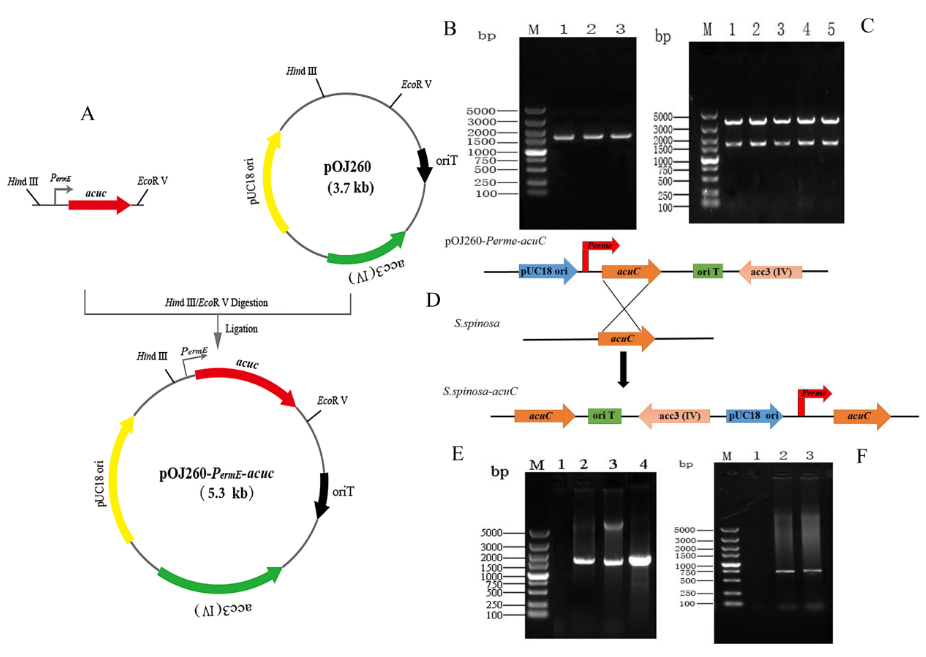


**Figure S2. Construction and verification of *S. spinosa*-*acuC***

A.Construction of the gene overexpression vector pOJ260-P_ermE_-*acuC*; B. The complete *acuC* gene fragment around 1.3 kb was ampified by using the genome of *S. spinosa* as the template and the P*_ermE_*-*acuC*-F and P*_ermE_*-*acuC*-R were used as primer pair; C. The vector pOJ260-P*_ermE_***-***acuC* was confirmed via restriction enzyme analysis and PCR amplification; D. The schematic diagram of *acuC* geneoverexpression; E. PCR validation of the *P_ermE_*-*acuC* fragment around 1.6 kb with the primer pair P*_ermE_*-F and P*_ermE_*-*acuC*-R; F. PCR validation of Apra resistance gene around 720 bp with the primer pair Apr*-*F and Apr*-*R.





**Figure S3.** The insecticidal activity of the wild-type and mutant strains on *H. armigera.*

*
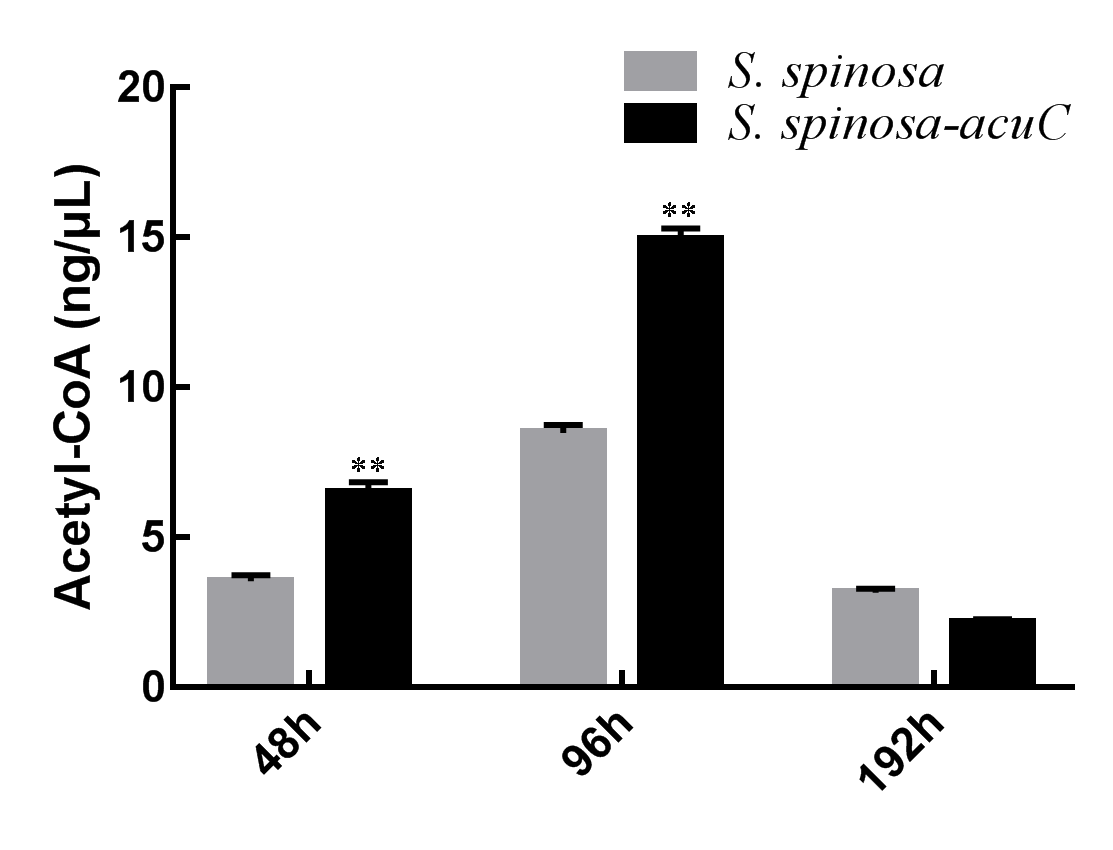
*

**Figure S4.** The content of Acetyl-CoA between the wild-type and overexpression strains in 48 h, 96 h and 192 h. Error bars represent the standard deviation of the mean. * and ** indicated *P<0.05* and *P<0.01*, respectively.


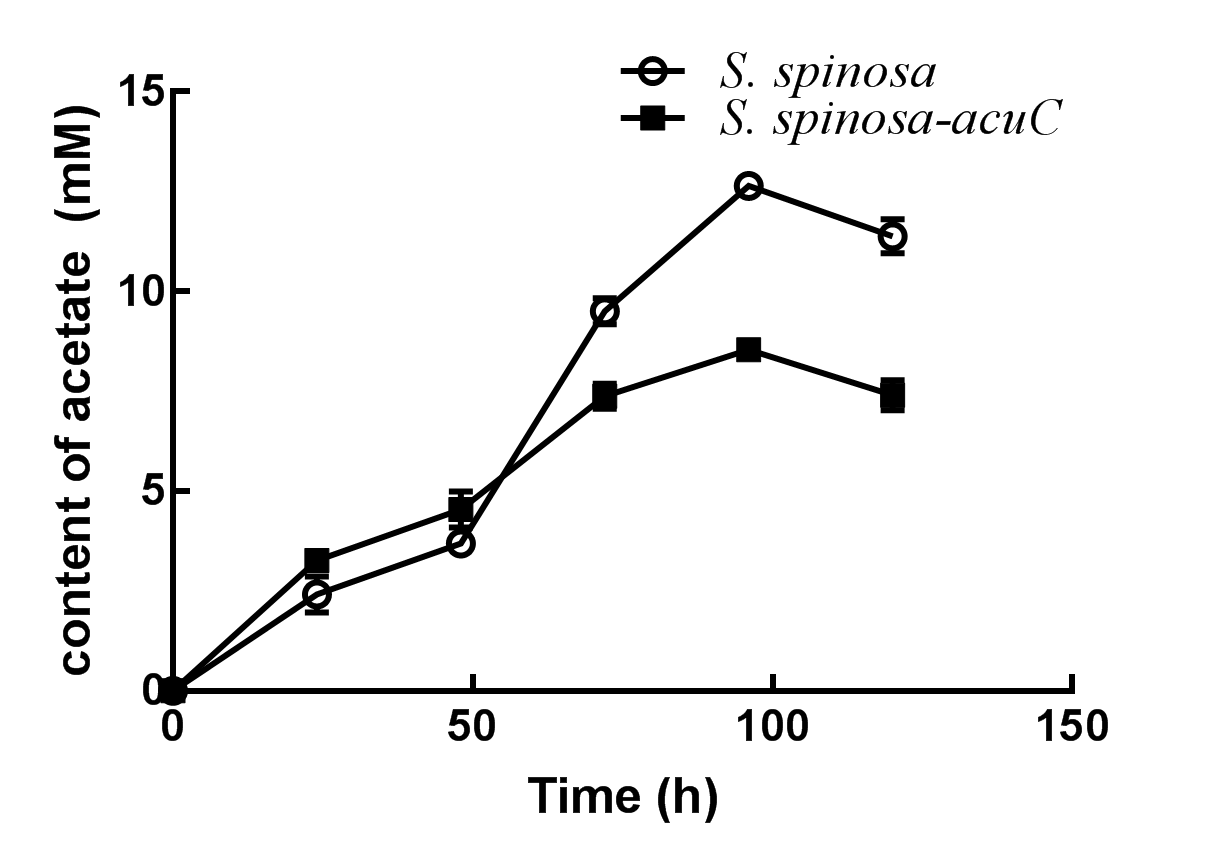


**Figure S5.** The acetate content remained in bacteria between the wild-type and overexpression strains during fermentation. Values are averages of triplicate bilogical samples. Error bars represent the standard deviation of the mean.


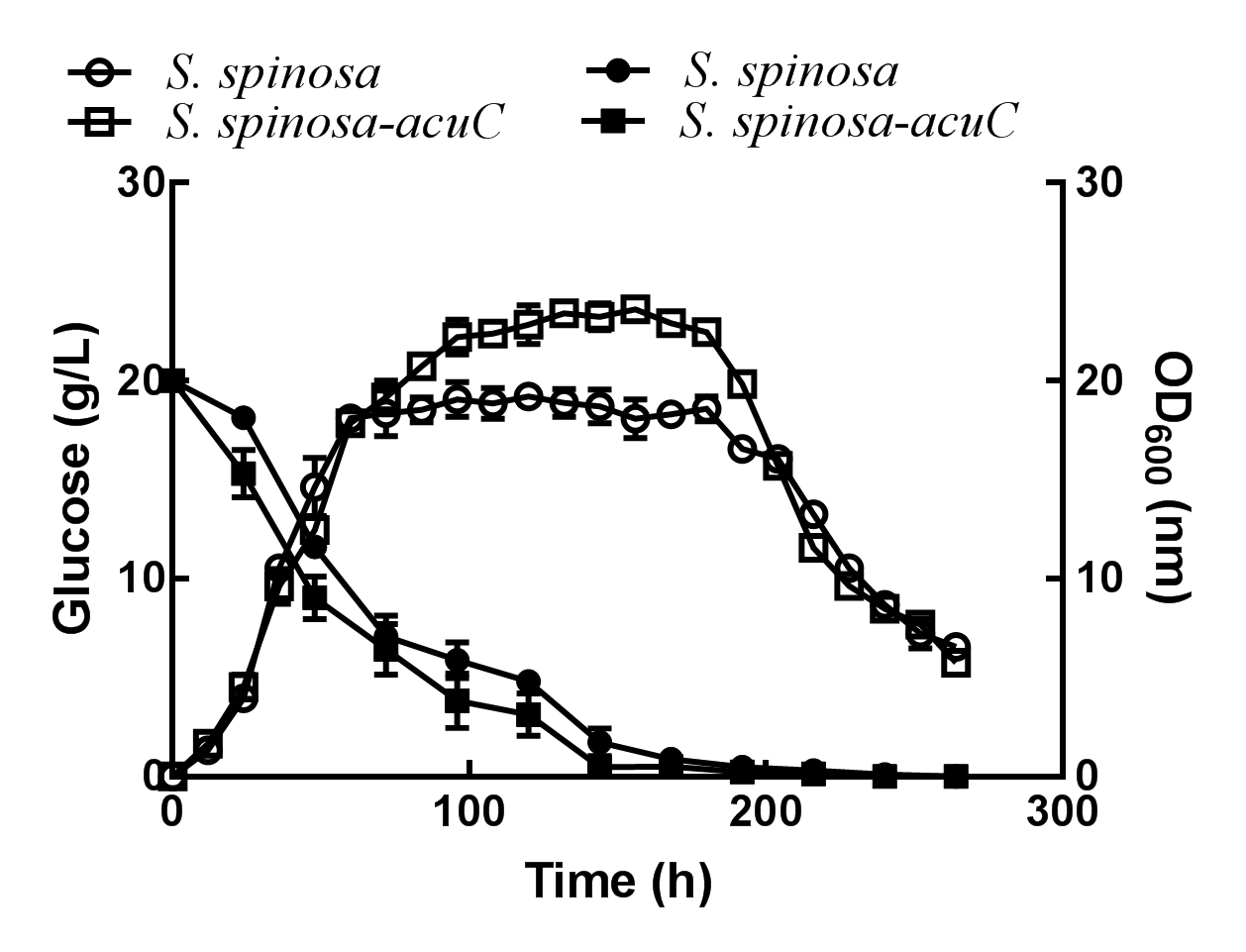


**Figure S6.** The consumption curve of glucose content in the fermentation medium between the wild-type and overexpression strains during fermentation. Values are averages of triplicate biological samples. Error bars represent the standard deviation of the mean.


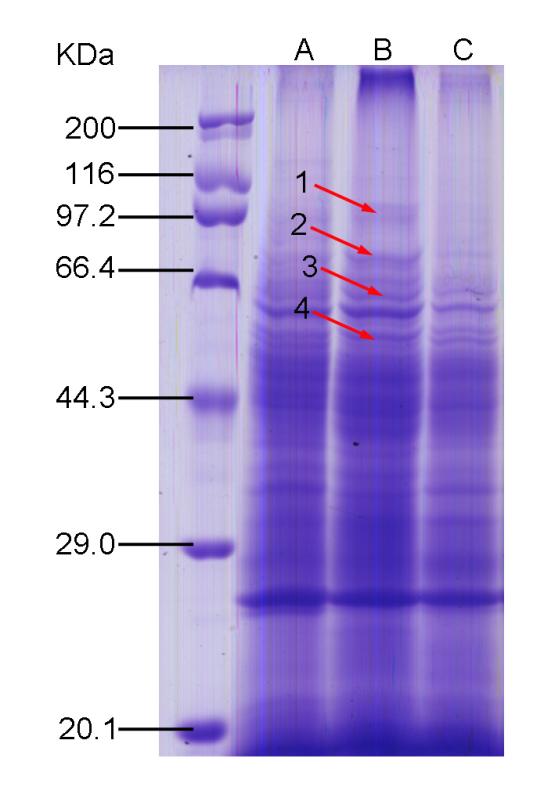


**Figure S7.** The SDS-PAGE analysis of whole-cell proteins of the wild-type and two mutant strains at 48 h. A. *S. spinosa*; B. *S. spinosa*-*acuC*; C. *S. spinosa*-Δ*acuC.*


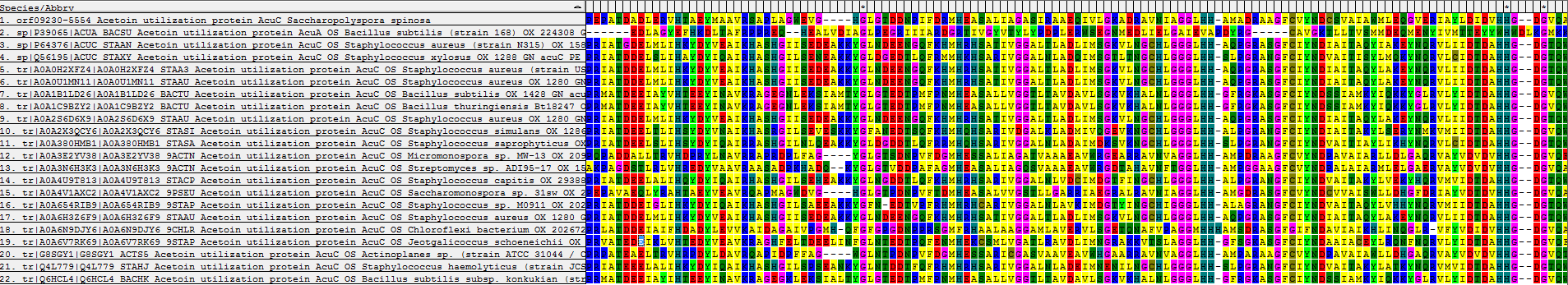


**Figure S8.** Comparison of acuC protein sequences among different bacteria. The same color indicated that the amino acid residues from the N-terminus of the proteins were conserved. 1. The acuC protein sequence in *S. spinosa*; 2-22. The acuC protein sequences of related proteins in different bacteria.
